# Villitis of unknown etiology is a placental pathology associated with pregnancy complication: a systematic review and meta-analysis

**DOI:** 10.3389/fmed.2025.1656438

**Published:** 2025-12-15

**Authors:** Xiaoqian Zhang, Kang Yan, Xietong Wang

**Affiliations:** 1 Key Laboratory of Maternal & Fetal Medicine of National Health Commission of China, Shandong Provincial Maternal and Child Health Care Hospital Affiliated to Qingdao University, Jinan, China; 2 Department of Obstetrics and Gynecology, Shandong Provincial Hospital, Shandong University, Jinan, China; 3 Department of Obstetrics and Gynecology, Shandong Provincial Hospital Affiliated to Shandong First Medical University, Jinan, China; 4 The Laboratory of Medical Science and Technology Innovation Center (Institute of Translational Medicine), Shandong First Medical University (Shandong Academy of Medical Sciences) of China, Jinan, China

**Keywords:** intrauterine growth restriction (IUGR), meta-analysis, preeclampsia (PE), stillbirth, villitis of unknown etiology (VUE)

## Abstract

**Background:**

Villitis of unknown etiology (VUE) is a chronic placental inflammatory lesion of high incidence, but its relationship with adverse pregnancy outcomes remains unclear. This meta-analysis quantifies its impact on five critical complications adverse pregnancy outcomes, including fetal growth restriction (IUGR), preeclampsia (PE), gestational hypertension, small gestation age (SGA), and stillbirth.

**Methods:**

We used the search verb “villitis of unknown etiology” OR “VUE” OR “chronic villitis” OR “unknown etiology of villitis”, from databases SCOUP, PubMed and Google Scholar, incorporating cohort studies, case–control studies. Heterogeneity was assessed via *I*^2^ statistics.

**Results:**

The incidence of VUE was higher in the IUGR, PE, gestational hypertension, SGA, and stillbirth groups than in the normal control group. VUE was significantly associated with an increased risk of stillbirth (OR = 3.64, 95% CI: 1.80–7.39) and pre-eclampsia (OR = 1.31, 95% CI: 1.03–1.65). A marginal association was observed between VUE and gestational hypertension (OR = 1.41, 95% CI: 0.892–2.23). In contrast, the associations of VUE with IUGR (OR = 1.56, 95% CI: 0.509–4.77) and SGA (OR = 1.01, 95% CI: 0.099–10.4) were not statistically significant and exhibited substantial heterogeneity.

**Conclusion:**

VUE is strongly associated with stillbirth and PE, highlighting its role in the pathogenesis of placental-related diseases. While there are no significant associations were found with intrauterine growth restriction or small for gestational age infants based on current evidence, these findings solidify the link between VUE and specific obstetric syndromes.

**Systematic Review Registration:**

https://www.crd.york.ac.uk/PROSPERO/display_record.php?RecordID=1077709, identifier PROSPERO (CRD4202121077709).

## Introduction

Villitis of unknown etiology (VUE) is a chronic placental inflammatory lesion characterized by lymphocytic and histiocytic infiltration of the chorionic villi, which persists after the exclusion of infectious etiologies. The incidence of VUE in term placentas is 5–15%, making it one of the common placental pathological changes (1). The pathological mechanism of VUE remains unclear, and it is currently believed to be associated with immune tolerance imbalance at the maternal-fetal interface, analogous to allograft rejection (2). VUE lesions can occur in either early or late pregnancy; however, their impact on placental development differs depending on the timing of onset. Furthermore, VUE lesions can be focal or diffuse, and the extent of involvement differentially affects placental function; besides, VUE can occur independently or frequently coexist with other inflammatory lesions, such as chronic histiocytic intervillositis, further compromising placental function (3). In summary, the impact of VUE on the placenta function is complex (4).

Pregnancy complication represents a significant public health issue in obstetrics, including stillbirth, intrauterine growth restriction (IUGR), small for gestational age (SGA), and preeclampsia (PE), gestational hypertension (5, 6). The global incidence of pregnancy complication remains high, severely affecting the health and quality of life of both mothers and perinatal infants.

Recent studies indicate that the incidence of PE was significantly higher in patients with VUE compared to the control group (7). Additionally, VUE has been associated with pathological changes such as placental insufficiency and abnormal umbilical artery blood flow (8). However, existing studies on the association between VUE and pregnancy complication suffer from limitations such as small sample sizes, the extended time span since publication and inconsistent findings (9). Effect sizes reported across different studies vary considerably, and conclusions regarding the strength of association with specific outcomes (e.g., preeclampsia, stillbirth) are inconsistent (10, 11). These limitations hinder the diagnosis and management of related conditions during pregnancy.

This study aims to systematically integrate existing research evidence, thereby increasing the sample size and enhancing statistical power to more accurately evaluate whether VUE was associated with IUGR, gestational hypertension, PE, preterm birth, and stillbirth. It will determine the strength of the association between VUE and various adverse pregnancy outcomes, ascertain the overall impact of VUE on pregnancy outcomes, and provide an evidence-based foundation for the clinical identification of high-risk pregnancies and the development of intervention strategies.

## Materials and methods

### Protocol registration and guidelines

This systematic review and meta-analysis were registered with PROSPERO (ID: 1077709) and conducted in accordance with the Preferred Reporting Items for Systematic Reviews and Meta-Analyses (PRISMA) guidelines. The Meta-analysis of Observational Studies in Epidemiology (MOOSE) checklist was also followed to ensure methodological rigor.

### Search strategy

A comprehensive literature search was performed across multiple databases, including Scopus and PubMed from the inception of the Amsterdam Placental Workshop Group Consensus in 2016 to 2023. The search strategy incorporated keywords as “(Chronic villitis) OR (Villitis of unknown etiology) OR (Chronic villitis of unknown etiology).” The search was restricted to human studies published in English, Spanish, or French. Additionally, gray literature, including conference proceedings and reference lists of relevant articles was manually searched to identify additional studies.

### Definition of terms

Conditions were defined according to standard clinical criteria and the definitions applied in the majority of included studies (12, 13). Preeclampsia (PE) was defined as new-onset hypertension (systolic BP ≥ 140 mmHg or diastolic BP ≥ 90 mmHg) occurring after 20 weeks of gestation accompanied by proteinuria and/or signs of maternal organ dysfunction (e.g., renal, hepatic, hematological, or neurological complications) or uteroplacental dysfunction. Gestational hypertension, also referred to as Gestational Hypertension, was defined as new-onset hypertension after 20 weeks of gestation in the absence of proteinuria or other features of preeclampsia. Intrauterine Growth Restriction (IUGR) was defined as a condition in which a fetus is unable to achieve its genetically determined growth potential, most commonly identified by ultrasonographic biometric measurements falling below the 10th percentile for gestational age and placental insufficiency (14). Small for Gestational Age (SGA) was defined as a birth weight falling below the 10th percentile for gestational age, based on population-based or customized growth standards, without implying an underlying pathological cause. Stillbirth was defined as fetal death occurring at or after 20 weeks of gestation (15).

### Study selection

**Figure 1:**
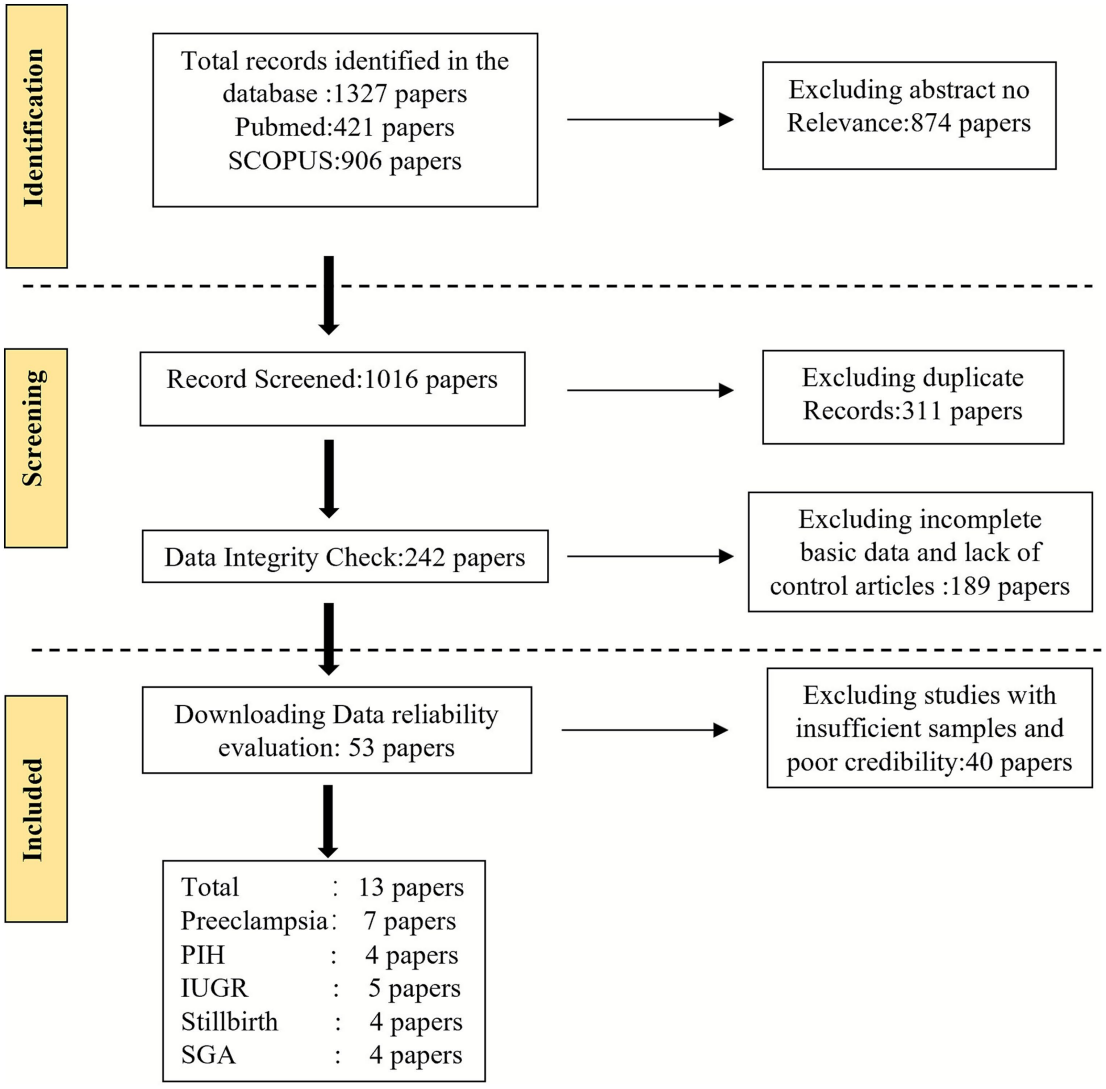
Flowchart for literature screening.

Two independent investigators screened titles and abstracts, followed by full-text assessment (Figure 1). Discrepancies were resolved through discussion or consultation with a third investigator.

*Inclusion criteria*: The cohort or case–control reporting placental VUE diagnosed according to the Amsterdam criteria. Studies providing data on perinatal outcomes, including intrauterine fetal growth restriction (IUGR), gestational hypertension, preeclampsia (PE), small gestational age (SGA) and stillbirth. Studies with a control group (e.g., pregnancies without VUE or with other placental lesions) for comparative analysis.

*Exclusion criteria*: Studies not adhering to the Amsterdam criteria for VUE diagnosis. Case reports, reviews, or studies without extractable outcome data. Studies involving multiple pregnancies, feticides, or placental lesions of infectious origin.

### Data extraction

Data were extracted using a standardized form, including: study characteristics (author, year, country, design, and sample size). Participant demographics (maternal age and BMI). Perinatal outcomes (IUGR, PE, stillbirth, gestational hypertension, and SGA). For studies with overlapping cohorts, the most comprehensive dataset was included. Authors were contacted for missing data where necessary.

### Quality assessment and risk of bias

The Newcastle-Ottawa Scale (NOS) was used to evaluate the quality of cohort and case–control studies, with scores ≥6 indicating good quality. The risk of bias was assessed across domains: selection (representativeness, exposure ascertainment), comparability (control for confounders), and outcome (assessment, follow-up). The Newcastle-Ottawa Scale assessment results for preeclampsia (PE), gestational hypertension, intrauterine growth restriction (IUGR), stillbirth, and small for gestational age (SGA) are detailed in Supplementary Tables S2, S3, S4, S5, and S6.

### Data synthesis and meta-analysis

Outcomes were summarized descriptively, stratified by VUE severity (low-grade vs. high-grade) and treatment interventions, if applicable. Dichotomous outcomes (e.g., stillbirth, IUGR) were pooled using odds ratios (ORs) with 95% confidence intervals (CIs). Heterogeneity was assessed using the *I*^2^ statistic (*I*^2^ > 50% indicating substantial heterogeneity). A random-effects model was applied if significant heterogeneity was present.

### Statistical analysis

RevMan 5.4 and R Studio (meta package) were used for meta-analysis. Statistical significance was set at *p* < 0.05. Where meta-analysis was not feasible, findings were synthesized narratively. This methodology ensures a rigorous and transparent approach to evaluating the association between VUE and adverse pregnancy outcomes.

## Results

### Incidence of villitis of unknown etiology (VUE) in pregnancy complication

The placental VUE related records in databases were screening and evaluated, then 13 studies were conformed to the inclusion criteria and were pooled for analysis (Supplementary Table S1).

The incidence of VUE across various obstetric syndromes is summarized in Table 1. Overall, VUE prevalence was consistently higher in pregnancies complicated by adverse outcomes compared to normal controls. Specifically, cases of preeclampsia (PE) demonstrated elevated median and mean VUE incidence relative to controls, despite considerable variation across studies. The most striking difference was observed in gestational hypertension, where the median VUE incidence in cases substantially exceeded that of controls. Similarly, both small for gestational age (SGA) and intrauterine growth restriction (IUGR) pregnancies showed notably higher VUE frequencies.

**Table 1:** Description of the incidence of pregnancy complication.

Pregnancy complication	Incidence of VUE (%)		
	Range	Median	Mean
	Case vs. normal	Case vs. normal	Case vs. normal
IUGR (2, 16–18)	18.9–50.0 vs. 8.0–50.0	39.4 vs. 22.4	39.9 vs. 23.4
PE (1, 2, 17, 19–22)	4.0–75.0 vs. 5.0–60.0	18.2 vs. 13.3	23.9 vs. 19.9
Gestational hypertension (1, 2, 19, 23)	0.6–40.4 vs. 0.8–34.5	14.3 vs. 5.0	15.2 vs. 11.3
Stillbirth (23, 29–31)	14.8–80.0 vs. 5.6–63.6	50.0 vs. 30.1	48.7 vs. 29.0
SGA (1, 2, 20, 23)	3.8–27.0 vs. 3.7–62.2	16.4 vs. 8.7	15.4 vs. 19.4

IUGR, intrauterine growth restriction; PE, preeclampsia; SGA, small for gestational age.

### VUE was associated with PE

**Figure 2:**
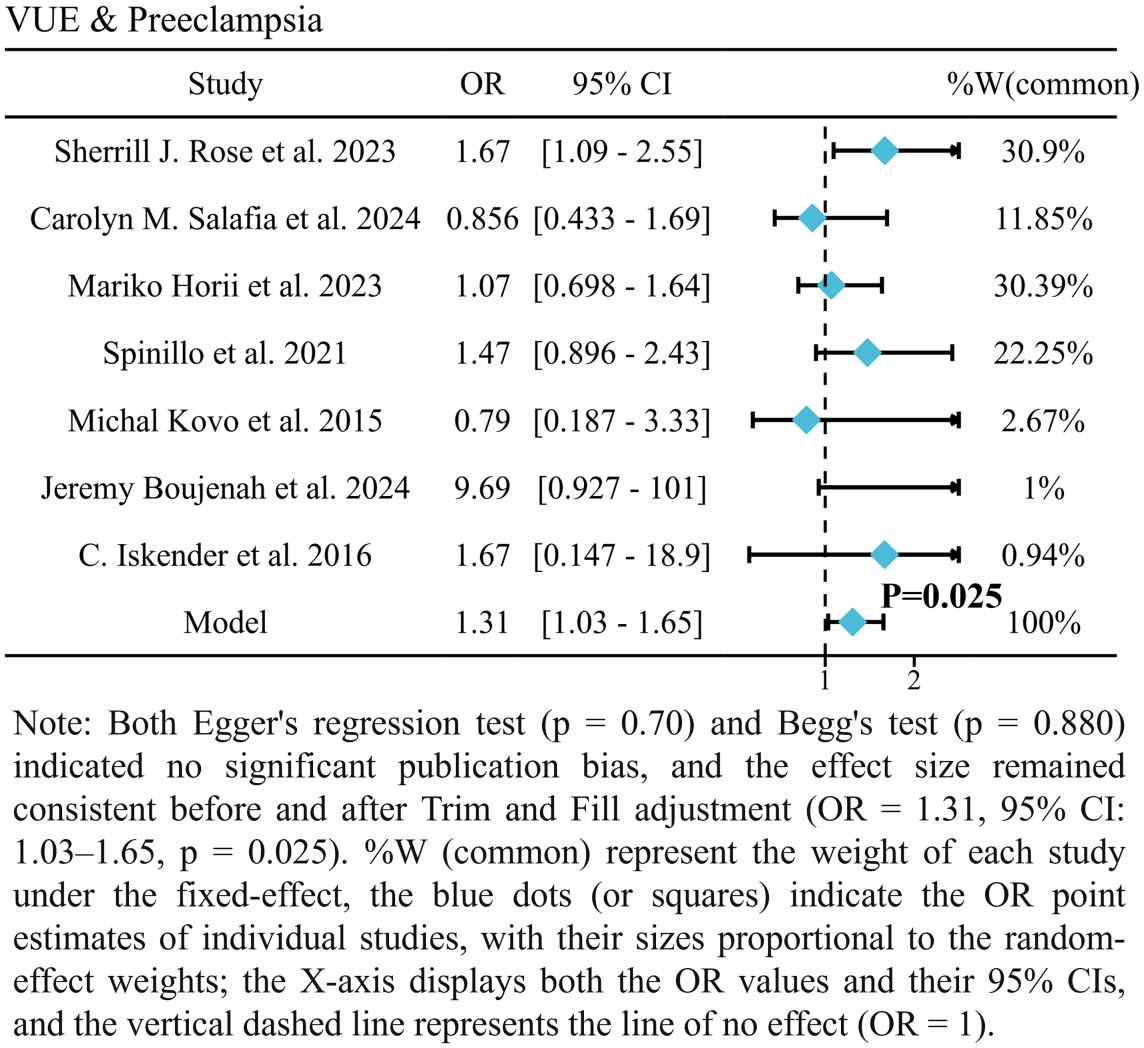
Forest plot of the association between VUE and preeclampsia.

Seven studies revealed a statistically significant increase in PE risk among VUE patients (OR = 1.31, 95% CI: 1.03–1.61, *p* = 0.025; Figure 2) (2, 7, 14, 16–19). This indicates 31% higher odds of developing PE in pregnancies complicated by VUE compared to controls. Additionally, there is low between-study heterogeneity among the enrolled studies (*I*^2^ = 16.1, 95% CI: 0–59.9%).

### VUE was associated with gestational hypertension

**Figure 3:**
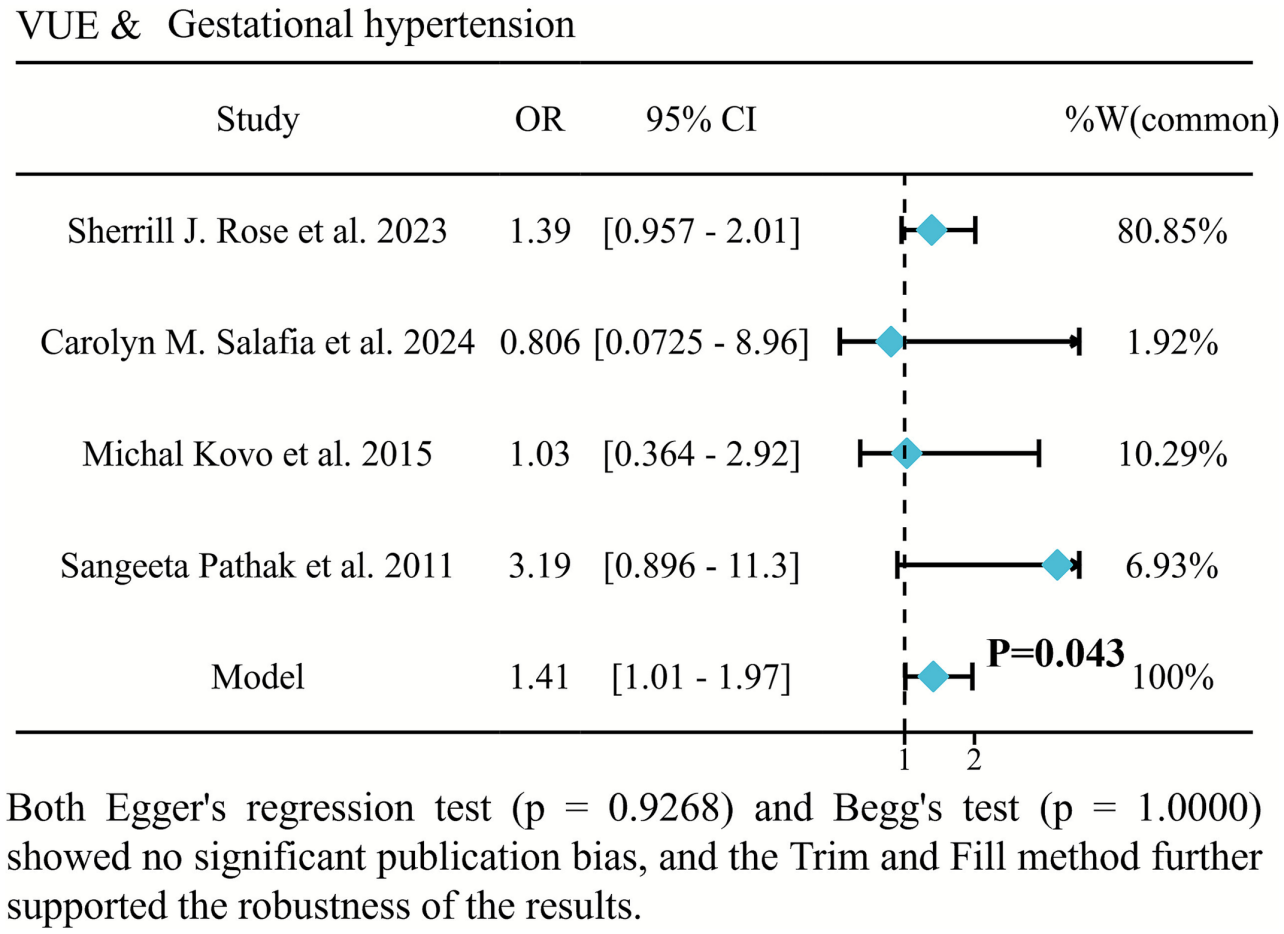
Forest plot of the association between VUE and gestational hypertension.

Four studies demonstrated a significant trend toward increased gestational hypertension risk in VUE-affected pregnancies (OR = 1.41, 95% CI: 1.01–1.97; *p* = 0.043; Figure 3) (2, 7, 16, 20). This indicates 41% higher odds of gestational hypertension in the VUE group, and the heterogeneity between the groups was minimal.

### VUE is not associated with IUGR

**Figure 4:**
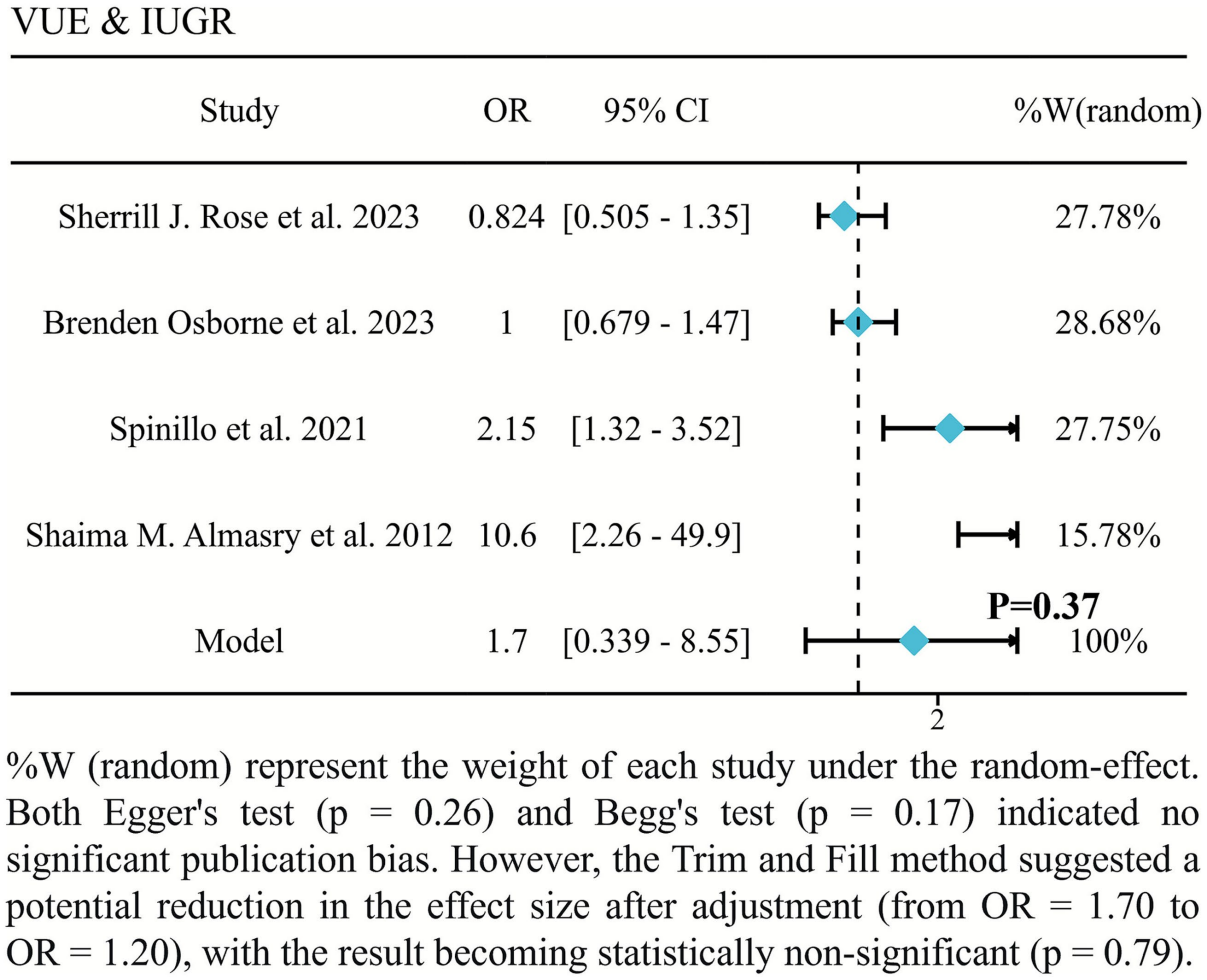
Forest plot of the association between VUE and UIGR.

The pooled analysis of the forest plot in Figure 4 showed no statistically significant association between VUE and IUGR (pooled OR = 1.70, 95% CI: 0.34–8.55, *p* = 0.37) (7, 13–15). Sensitivity analysis using the Trim and Fill method showed a reduction in the effect size that remained statistically non-significant after adjustment (adjusted OR = 1.20, *p* = 0.79).

### VUE was associated with stillbirth

**Figure 5:**
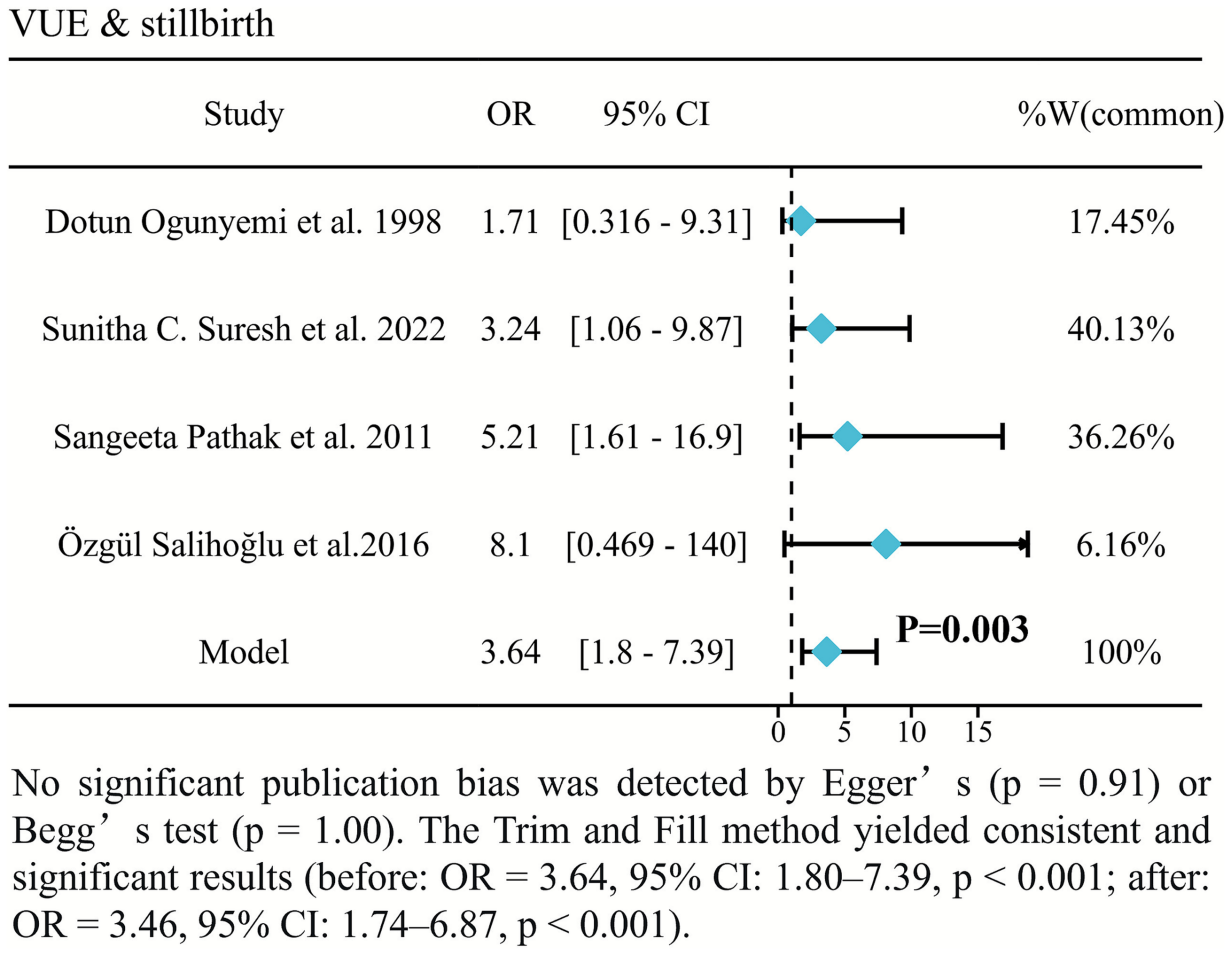
Forest plot of the association between VUE and stillbirth.

A robust and statistically significant association was found between VUE and stillbirth (OR = 3.97, 95% CI: 2.02–7.79, *p* < 0.0001; Figure 5) (20–23). VUE-associated pregnancies exhibited nearly 4-fold higher odds of stillbirth, with remarkable homogeneity across studies (*I*^2^ = 0%).

### VUE is not associated with SGA

**Figure 6:**
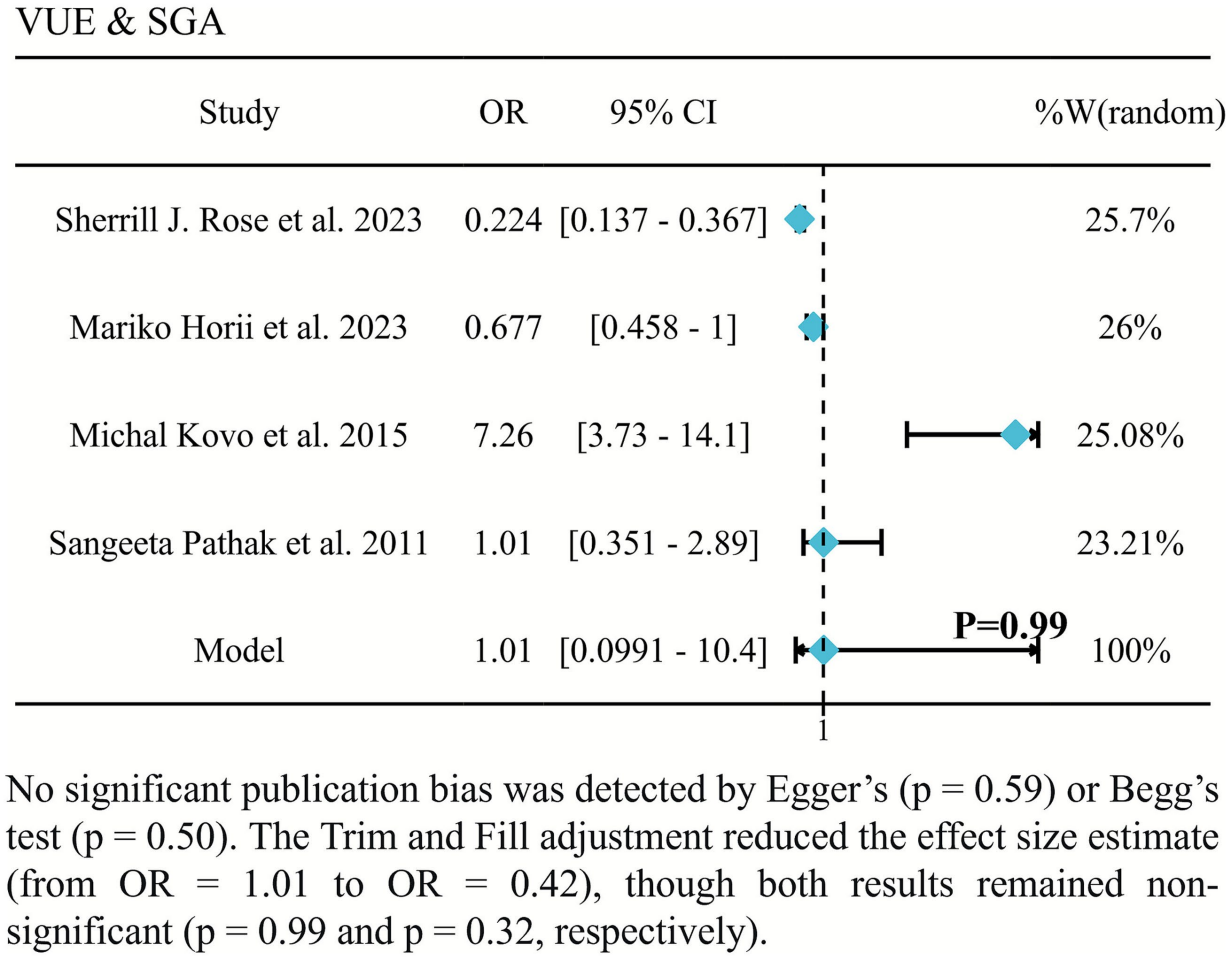
Forest plot of the association between VUE and SGA.

The association between VUE and SGA was not statistically significant (OR = 1.61, 95% CI: 0.44–5.90; Figure 6) (2, 7, 17, 20). Substantial heterogeneity was observed among the included studies (*I*^2^ = 85.1%).

## Discussion

This meta-analysis, incorporating seven studies of high methodological quality (Newcastle-Ottawa score >7; Supplementary Table S2), demonstrates that villitis of unknown etiology (VUE) was associated with a 1.31-fold increased risk of preeclampsia (PE). Our analysis synthesized using a common-effects meta-analysis with Hartung-Knapp adjustment, establishes VUE is significantly associated with PE. This study provides robust evidence establishing a significant association between VUE and PE, offering novel insights into the underlying mechanisms of this condition.

Regarding IUGR, our Hartung-Knapp adjusted random-effects meta-analysis of eight studies no statistically significant association with intrauterine growth restriction, appears to contradict the conclusions of Derricott et al.’s (24) systematic review, which suggested a clearer contributory role of VUE in IUGR pathogenesis. This discrepancy underscores the need for further investigation to clarify the nature of this relationship.

For gestational hypertension, our fixed-effects meta-analysis of four studies, which showed minimal heterogeneity, demonstrated a statistically significant yet borderline association with VUE. It should be noted that this result was largely influenced by Sherrill’s study comprising over 80% of the weight, and the confidence interval narrowly crossed the null threshold. Therefore, while a positive association is suggested, this finding should be interpreted with caution and warrants validation through future studies with larger sample sizes and more balanced study contributions.

Our Hartung-Knapp adjusted fixed-effects meta-analysis of five studies revealed that VUE was associated with a marked, nearly 4-fold increase in the odds of stillbirth. This robust association, which is consistent with other systematic reviews such as that by Narice et al., underscores VUE was associate with fetal demise (25).

For SGA, our random-effects meta-analysis of five studies showed no significant association. The observation should careful interpretation. A critical methodological consideration is that the included SGA studies did not adequately adjust for the confounding effect of gestational age. Preterm infants inherently carry a higher risk of being classified as SGA compared to term infants (26). Given that VUE predominantly manifests during third trimester, preterm SGA infants delivered before the typical onset of VUE would likely test negative for VUE, despite their growth restriction (27). This potential misclassification bias may have attenuated the observed association between VUE and SGA status in this analysis.

### Study limitations

The number of studies eligible for inclusion in this analysis was limited, with only four robust studies available for gestational hypertension, SGA, and IUGR. Therefore, the conclusions and interpretation of these results must be approached with caution. Additionally, although no significant publication bias was detected among the included studies, all placental samples were derived from hospital-based populations, inevitably introducing Berkson’s bias and selection bias. Additionally, VUE frequently co-occurs with other placental inflammatory lesions, such as chronic histiocytic intervillositis or chronic chorioamnionitis (28). The pathological assessment in the included studies often did not distinguish between isolated VUE and VUE occurring alongside these other conditions. This lack of discrimination between pure and mixed VUE pathologies may confound the estimation of the specific contribution of VUE to the studied adverse outcomes.

## Conclusion

In conclusion, this systematic review and meta-analysis provide robust evidence establishing villitis of unknown etiology (VUE) as a significantly associated with adverse pregnancy outcomes, notably preeclampsia and stillbirth. Although the available data cannot definitively determine the impact of mixed inflammatory lesions, the consistency of our findings across multiple studies underscores the clinical relevance of VUE in the spectrum of placental-related diseases. The associations identified reinforce the role of maternal-fetal immune dysregulation as a potential shared mechanism. Future research should prioritize standardized VUE grading, distinguish isolated from mixed inflammatory lesions, and explore the predictive value of VUE for targeted monitoring and management strategies in high-risk pregnancies.

## Data Availability

The original contributions presented in the study are included in the article/Supplementary material, further inquiries can be directed to the corresponding authors.
